# Involuntary closures of for-profit care homes in England by the Care Quality Commission

**DOI:** 10.1016/S2666-7568(24)00008-4

**Published:** 2024-04

**Authors:** Anders Bach-Mortensen, Benjamin Goodair, Michelle Degli Esposti

**Affiliations:** a Department of Social Policy and Intervention, University of Oxford, Oxford, UK; b Department of Social Sciences and Business, Roskilde University, Roskilde, Denmark; c Institute for Firearm Injury Prevention, University of Michigan, Ann Arbor, MI, USA

## Abstract

Adult social care services in England are struggling, and sometimes failing, to supply the quality of care deserved by the most vulnerable people in society. The Care Quality Commission (CQC) is responsible for protecting the recipients of this crucial public service. Their strongest enforcement is the ability to cancel the registration—the legal right to operate—of a health or social care provider. Using novel data from the CQC, we show that the proportion of care home closures due to CQC enforcements, relative to all closures, is increasing. Since 2011, 816 care homes (representing 19 918 registered beds) have been involuntarily closed by the CQC. Our results show that effectively all involuntary closures (804/816) occurred in for-profit care homes. This data emphasises the need for a comprehensive assessment of the impact of for-profit provision on the quality and sustainability of adult social care in England.

## Introduction

Social care services in England are on the brink of a crisis. Underfunded, understaffed, and struggling to supply the quality of care deserved by the most vulnerable people in society.^1^, ^2^ Unmet need is increasing^3^, ^4^ and reports consistently diagnose the sector as fragile and unsustainable.^1^, ^4^, ^5^, ^6^ In this tough environment, residents must be protected against receiving poor quality care.

In England, adult social care is operated in a two-tier system composed of self-funded and state-funded residents. Residents are eligible for state support if their savings do not exceed £23 250 or if they do not own their home—although this limit is scheduled to change in October, 2025 to allow savings of up to £100 000.^7^ Most care homes serve a mix of state-funded and self-funded residents, and less than 1·3% of all care homes only include self-funded residents.^8^ However, funding for state-funded residents is widely considered inadequate,^6^, ^9^ and the ability to attract self-funders has increasingly become financially imperative.^10^ Inadequate public funding has created an incentive for providers to focus on access to self-funders rather than care needs, increasing the risk of unmet and under-met need for residents who are already vulnerable.^11^ Little is known about the specific characteristics of self-funders versus state-funders, but there is a clear tendency that care homes operating in less deprived areas have a higher proportion of self-funders.^12^

The care crisis has corresponded with an increase in private provision of care services, including both third sector (non-profit) and for-profit services. Most care homes in England are operated on a for-profit basis, which is not a new development. The private (but primarily for-profit) sector has played a key role in care home provision since the National Health Service (NHS) and Community Care Act in 1990,^13^ and the market share of the for-profit sector has steadily increased over time (appendix p 5). More than 85% of all care homes and 88·7% of registered beds in England in September, 2023, were operated by for-profit providers.

The challenges facing adult social care provision are not unique to England, and neither is its attempt to solve them via marketisation—a term used to refer to the increased use of market-based competition in purchasing, organising, and regulating of public services. Many countries are struggling to finance increasing care demands with market mechanisms to optimise quality and efficiency and to increase the number of providers in the sector.^14^ The trend towards outsourcing social care is evident in numerous EU and Organisation for Economic Co-operation and Development countries, where private provision is on the rise.^15^, ^16^

As the independent regulator of health and social care in England, the Care Quality Commission (CQC) is responsible for protecting the recipients of this crucial public service. Their primary function is to inspect health and social care providers and assess and report the quality of these services. The current inspection framework for adult social care was introduced in 2014 and involves assessing services on the basis of five domains—whether a service is safe, effective, caring, responsive, and well led—as well as an aggregated overall rating.^17^, ^18^ Each domain can be rated inadequate, requires improvement to be good, good, or outstanding (appendix pp 1–3). The CQC aims to inspect a care home within 12 months of the care home registering with them. The subsequent inspection frequency depends on the inspection rating—care homes rated outstanding will normally be inspected within 5 years and homes rated good within 3·5 years, whereas homes will be inspected within 2 years if rated requires improvement to be good or within 1 year if rated inadequate.^19^ Homes that are continuously rated inadequate will be put into special measures, which involves more frequent visits and closer scrutiny (panel).

Panel **Enforcements leading to involuntary closure**

The Care Quality Commission (CQC) can cancel a registration as a sanction for care homes that do not adhere to their requirements and safety standards. The CQC will only consider cancelling a registration if people receiving care either have “…suffered harm or are at risk of harm because a registered person is failing to comply with legal requirements” or are “receiving care services that substantially fail to meet the standards set out in the regulations”.^20^

A cancellation will typically only be considered after significant efforts to ensure compliance with legal requirements, and care homes that have been rated as inadequate will typically be put into special measures,^21^ which involves more frequent scrutiny and inspection. However, an enforcement can be implemented with immediate effect if the risk of harm is acute or in instances of severe safety and regulation breaches. If providers continue to operate after their registration has been cancelled, it will be considered a criminal offence.^20^

In addition to publishing inspection reports, one of the strongest enforcements available to the CQC is their ability to cancel the registration—the legal right to operate—of a health or social care provider. Cancelling a care home's registration is one of the strongest regulatory powers held by the CQC (panel). It serves as a last resort for care homes that have put their residents at risk or that continuously do not live up to CQC standards.

Despite the significance of these events, little is understood about how the CQC uses this power, if it has changed over time, and what type of care homes are most commonly closed because of an enforcement. Using novel CQC data on adult care homes, we show that involuntary closures are on the rise and happens almost exclusively to for-profit homes.

## Data sources

To map the voluntary and involuntary closures over time, we harmonised data from various sources of CQC data. We received a comprehensive list of CQC-enforced involuntary closures, 2011–23 (as of September), directly from the CQC. Data on the care home and client characteristics of registered and deregistered homes were retrieved from the publicly available CQC data repository.^22^ We used data from the CQC application programming interface (API)^23^ to identify care home closures over time. We were able to connect this data with the location identity, which is the unique CQC identifier for registered health and social care providers.

We coded care home ownership by categorising all registered charities and charitable companies as third sector, and all private companies, partnerships, and individual providers without a charity number as for-profit. All council and NHS trust care homes were coded as public.

In this Health Policy, we focus on involuntary closures and voluntary closures that were not due to a provider takeover. We used the location ID to distinguish between different types of care home closures. A care home location ID will change if the care home closes, changes owner, or changes address. As such, a change in location ID does not necessarily mean that the care home will cease to operate. We used data from the CQC API, which tracks the life course of care homes across location ID and enables us to identify closures (in the sense of the care home ceasing to operate) from address changes and provider takeovers.

## Involuntary care home closures

**Figure 1:**
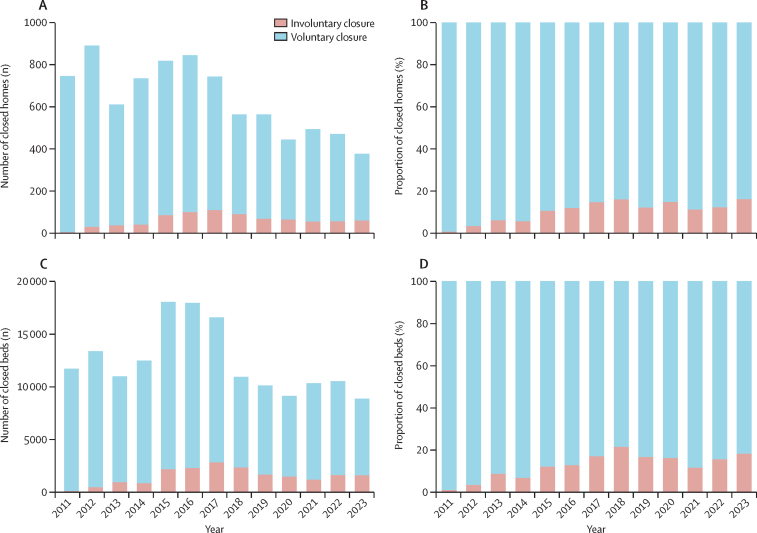
Number and proportion of involuntary and voluntary closures of closed homes and beds since 2011 (A) Number of closed homes. (B) Proportion of closed homes that are due to involuntary or voluntary closure. (C) Number of closed beds. (D) Proportion of closed beds that are due to involuntary or voluntary closure. Data sources: Care Quality Commission data on voluntary and involuntary closures and publicly available registration data.^22^ Year 2023 is as of September, 2023.

816 involuntary care home closures (representing 19 918 registered beds) were recorded from January, 2011, to September, 2023 (figure 1). Concerningly, the proportion of involuntary to voluntary care home closures is increasing. 61 (16%) of 377 care home closures in England in 2023 (as of September) were because of an enforcement. This is an increase of more than 10 percentage points compared with 2012 and 2011 (appendix p 4). The impact of involuntary closures is even higher when looking at the proportion of registered beds (figure 1C, D). 1657 (15·7%) of 10 521 closed beds in 2022, and 1630 (18·4%) of 8878 closed beds in 2023 (as of September), were the result of an involuntary closure.

The number and percentage of involuntary care home closures increased sharply in 2014 following the change in the CQC inspection framework,^24^ suggesting a change in enforcement practice (figure 1). However, the degree to which this trend in involuntary closures represents a change in provider performance, CQC enforcement practices, or a combination of both, is unclear. What is indisputable, however, is that a registration cancellation signifies a grave breach of regulations and a substantial risk to the safety of residents. The CQC exercises this authority judiciously, reserving it for care home providers it deems incapable of substantially complying with regulations or those deemed likely to fail in doing so.^20^

## Care home closure by enforcement

We linked the novel care home closure data to CQC registration data to explore the characteristics of care homes that have been closed voluntarily or through enforcement, as well as those that were active as of September, 2023. Nearly all care homes that have been subject to an involuntary closure were run on a for-profit basis (table). From January, 2011 to September, 2023, 816 involuntary care home closures (representing 19 918 registered beds) were recorded, and all except for 12 closures happened to for-profit care homes. 507 (62·1%) of 816 care homes were registered as companies. Individual and partnership organisations accounted for 297 (36·4%) of 816 involuntary closures. 424 (52·0%) of 816 involuntary closures happened to care homes that were registered to work with patients with dementia.

**Table:** Care home characteristics of involuntary and voluntary closed and active care homes

	**Involuntary closures, 2011–23**	**Voluntary closures, 2011–23**	Active care homes*, September 2023
**Ownership**			
For-profit	804/816 (98·5%)	6086/8299 (73·3%)	12 581/14 729 (85·4%)
Public	2/816 (0·2%)	670/8299 (8·1%)	402/14 729 (2·7%)
Third sector	10/816 (1·2%)	1543/8299 (18·6%)	1746/14 729 (11·9%)
**Organisation type of for-profit homes**†			
For-profit company	507/816 (62·1%)	4384/8299 (52·8%)	11 520/14 742 (78·1%)
Individual or partnership	297/816 (36·4%)	1701/8299 (20·5%)	1069/14 742 (7·3%)
**Client characteristics**			
Disabled	243/816 (29·8%)	2200/8299 (26·5%)	5862/14 742 (39·8%)
Mental health needs	219/816 (26·8%)	1862/8299 (22·4%)	4064/14 742 (27·6%)
Detained under Mental Health Act	11/816 (1·3%)	109/8299 (1·3%)	135/14 742 (0·9%)
Dementia	424/816 (52·0%)	2813/8299 (33·9%)	7662/14 742 (52·0%)
**Care home characteristics**			
Includes nursing‡	200/816 (24·5%)	1489/8299 (17·9%)	4068/14 729 (27·6%)
Months of registration, mean (SD)	72·47 (36·49)	56·48 (40·58)	116·22 (49·84)
Care home beds, mean (SD)	24·41 (17·68)	19·42 (20·16)	30·81 (25·29)
**Latest overall rating**			
Inadequate	442/582 (75·9%)	814/3967 (20·5%)	174/14 291 (1·2%)
Requires improvement	88/582 (15·1%)	992/3967 (25·0%)	2456/14 291 (17·2%)
Good	52/582 (8·9%)	2144/3967 (54·0%)	11 051/14 291 (77·3%)
Outstanding	0	13/3967 (0·3%)	609/14 291 (4·3%)
Missing inspection data	234/816 (28·7%)	4332/8299 (52·2%)	459/14 750 (3·1%)

Data are n/N (%) unless otherwise stated. Data sources: Care Quality Commission data on voluntary and involuntary closures and publicly available registration data.^22^ This table does not count the voluntary closures that are due to a provider takeover.

* The denominator for active care homes varies due to missing data on some variables.

† Private companies, partnerships, and individual providers without a charity number were coded as for-profit.

‡ A home was coded as a nursing home if it was registered as a care home service with nursing.

442 (54·27%) of all 816 involuntary closed care homes and 442 (75·9%) of 582 involuntary closures with any rating from the inspection framework were rated as inadequate. 234 (28·7%) of 816 homes that were closed by the CQC did not have an inspection rating, either because the home had not received a new inspection rating following the 2014 change in inspection framework or if the care home was closed before receiving an inspection rating. Most involuntary closures without an inspection rating occurred before 2018 (appendix p 5).

News items from both the CQC and sector outlets corroborate instances of the CQC closing care homes that were repeatedly rated inadequate,^25^, ^26^ which put residents at risk of harm.^27^, ^28^, ^29^ Notably, 140 (24·1%) of 582 care homes with a CQC rating that were closed via an enforcement did not have an inadequate rating, and 52 (8·9%) of 582 homes were even rated good. According to the CQC's enforcement policy,^20^ this closing of homes rated as higher than inadequate suggests instances of urgent enforcement due to extreme and acute risk of harm.^30^ For example, a care home in Barnsley was closed with immediate effect in September, 2023, due to urgent safety concerns, even though its last rating was requires improvement to be good.^31^

**Figure 2:**
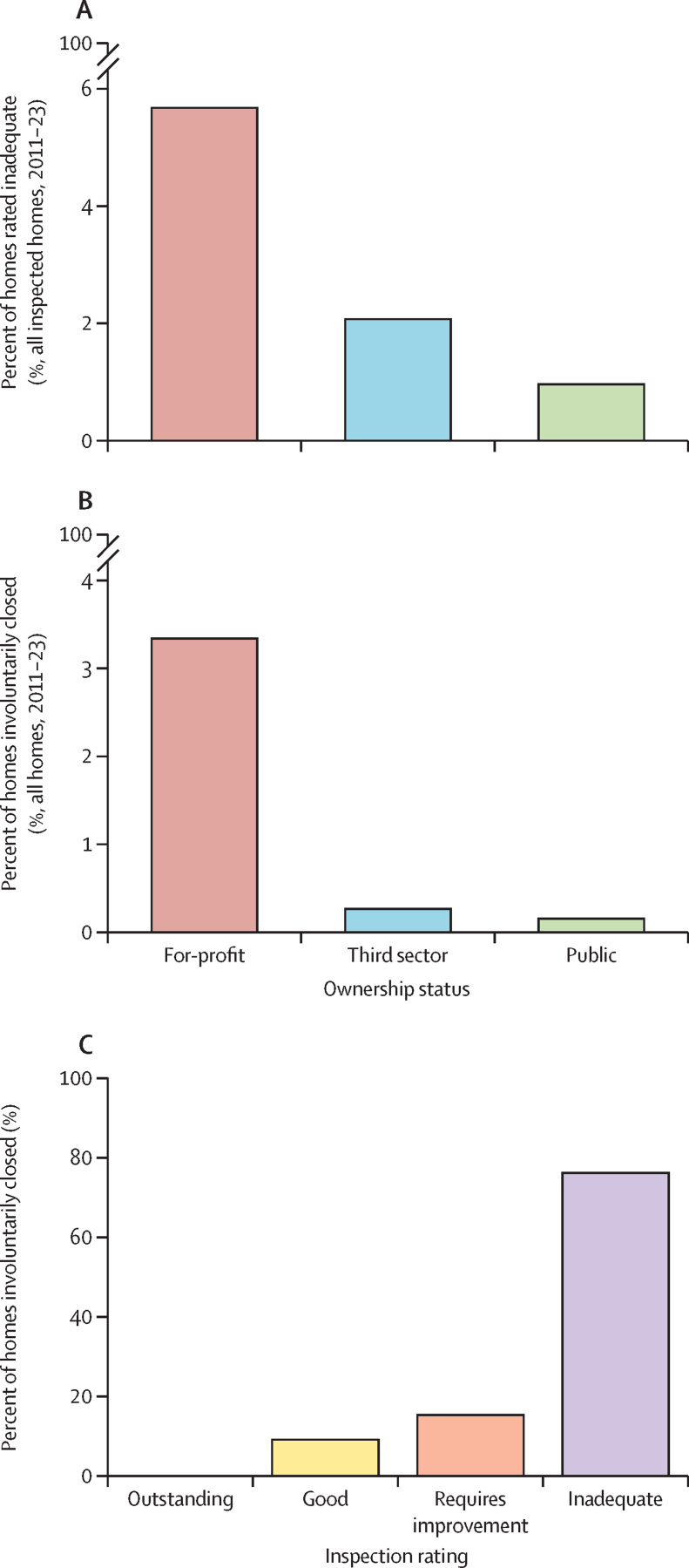
Characteristics of involuntarily closed and inadequately rated homes (A) The proportion of homes that are rated inadequate in each sector. (B) The proportion of homes that are involuntarily closed in each sector. (C) The last inspection rating of involuntarily closed homes.

1025 (5·7%) of 18 051 for-profit homes were rated inadequate in their latest inspection (of those with a rating, including active and closed homes), whereas 6 (1·0%) of 628 public sector homes and 58 (2·1%) of 2805 third sector homes were rated inadequate (figure 2). 804 (3·31%) of 24 278 for-profit homes in 2011–23 have been closed by the CQC versus 2 (0·15%) of 1327 public sector homes and 10 (0·26%) of 3832 third sector homes (figure 2B). This statistic translates into one in 30 for-profit care homes having been closed involuntarily.

## Implications for adult social care

In England, there is broad agreement that the adult social care sector is struggling and underfunded—representing a care crisis.^2^, ^4^, ^32^, ^33^ Concerns about the state of the sector intensified following the 2010 budget cuts, after which public funding declined and failed to keep pace with the development increase in people in need of care.^32^, ^34^ The inadequacy of social care was further highlighted by the severe impact of the pandemic among social care residents.^35^, ^36^ Moreover, a 2018 Competition and Markets Authority investigation of the adult social care market found that the financing model is unsustainable and more public funding is urgently needed. This concern has been echoed by the CQC itself, which has continuously raised concerns around the fragility of the sector and warned that the absence of a sustainable funding plan poses a serious risk to the sector and its most vulnerable residents.^1^, ^5^ The 2022 and 2023 increases in inflation have further exposed the financial vulnerability of homes relying on public funding, leaving state-funded residents at increased risk of inadequate or unmet care.^37^

There is contentious debate about whether marketisation and private sector involvement has worsened the crisis facing adult social care. Although there is a consensus that the sector is underfunded and understaffed, opinions diverge on the role of the private sector. Some people argue that private sector principles are ill-suited to the nature of care work, whereas others contend that marketisation is essential for the future of the sector.^38^

Marketisation is grounded on the core assumption that the market is responsive to care quality, suggesting that competition and user choice in the tender process allows the best homes to operate. This assumption has been extensively challenged due to, among other things, the vulnerability of the client group (often people who are older and frail), reduced budgets for state-funded residents, and inadequate regulatory levers to tame private provision among local authorities and the CQC.^38^, ^39^ This scepticism is largely supported by the academic literature; research examining care home ownership and quality indicates that care homes operated under for-profit models, including chain ownership and private equity involvement, tend to perform worse than third and public sector provision.^40^, ^41^, ^42^, ^43^ In England, research further suggests that although competition in the care home sector led to lower prices, it also pushed quality standards towards minimal requirements.^44^ Similar trends have been observed in other countries,^45^, ^46^ and the literature on health-care marketisation consistently supports the idea that competition rarely enhances quality of care.^47^, ^48^

Involuntary closures are absent from the ownership debate and research. Although they are rare events, they represent serious regulation and safety breaches that will typically involve harm to clients. A care home closure is also likely to cause substantial costs to both the local authority and clients in need of relocation.^49^ More importantly, such events can be traumatic and harmful for already vulnerable residents. Notwithstanding the extremity of these events, involuntary closures should therefore be tracked and evaluated to identify trends and risk factors. The data in this paper reveal that nearly all involuntary closures happened to for-profit care homes. To protect residents going forward, exploring if the reason that enforced closures occur exclusively to for-profit care homes is systematic or coincidental is key.

## Conclusion

The data on involuntary closures give us additional insight on the provision of social care by highlighting a significant and concerning trend in the involuntary closures of care homes in England. It shows a noteworthy increase in enforcements in for-profit care homes, particularly following the implementation of new quality standards in 2014. This development is concerning; it suggests that around 20 000 residents have been forced to relocate urgently due to severely inadequate care.

The dominance of for-profit providers in the care home industry and their disproportionate association with enforcement actions raise questions about the role of the private sector in exacerbating the sector's ongoing crisis. Research has already indicated that for-profit care homes tend to perform worse and that competition in the sector might not improve quality. As the debate about the future of the sector continues, these data emphasise the need for a comprehensive assessment of the impact of for-profit provision on the quality and sustainability of adult social care in England.

## Data sharing

All the data used in this manuscript and reproducibility materials to repeat the analysis are openly available at https://github.com/BenGoodair/Involuntary_care_home_closures.

## Declaration of interests

We declare no competing interests.
