# Supplementary material for: Involuntary closures of for-profit care homes in England by the Care Quality Commission
Source: Lancet Healthy Longev. 2024 Apr;5(4):e297–302. doi: 10.1016/S2666-7568(24)00008-4 (PMC11649842; doi:10.1016/S2666-7568(24)00008-4)
Supplement: Supplementary appendix [file mmc1.pdf]

THE LANCET
Healthy Longevity

Supplementary appendix

This appendix formed part of the original submission and has been peer reviewed. We post it as supplied by the authors.

Supplement to: Bach-Mortensen A, Goodair B, Degli Esposti M. Involuntary closures of for-profit care homes in England by the Care Quality Commission. *Lancet Healthy Longev* 2024; published online March 12. https://doi.org/10.1016/S2666-7568(24)00008-4.

Supplementary material

Table A1: Inspection domains – adapted from (1) and (3)

**Domain**	
Safe	"By safe, we mean people are protected from abuse and avoidable harm. Abuse can be physical, sexual, mental or psychological, financial, neglect, institutional or discriminatory abuse"
Effective	"By effective, we mean that people's care, treatment and support achieves good outcomes, promotes a good quality of life and is based on the best available evidence" (1)
Caring	"By caring, we mean that the service involves and treats people with compassion, kindness, dignity and respect." (1)
Responsive	"By responsive, we mean that services meet people's needs." (1)
Well-led	"By well-led, we mean that the leadership, management and governance of the organisation assures the delivery of high-quality and person-centred care, supports learning and innovation, and promotes an open and fair culture." (1)
Overall	Overall location ratings refer to an aggerate measure which are determined by the following principles: First, if there is a breach of regulations, the highest overall rating possible is 'requires improvement'. Second, all five key questions hold equal weight. Third, to achieve an 'outstanding' rating, at least two questions must be rated as 'outstanding,' while three should be rated as 'good'. Fourth, various combinations can lead to a 'good' rating; typically, this involves no more than one question rated as 'requires improvement' and none as 'inadequate'. Fifth, if two or more questions 'require improvement,' the overall rating usually becomes 'requires improvement.' Sixth, if two or more questions are rated as 'inadequate,' the overall rating typically becomes 'inadequate'. (3)

More details about how the CQC inspects each domain can be found here (1).

Table A2: Rating characteristics – adapted from (4)

**Safe**			
Outstanding	***Good***	***Requires improvement***	***Inadequate***
"People are protected by a strong, empowering and distinctive approach to safety and a focus on openness, transparency and learning when things go wrong."	"People are protected from avoidable harm and abuse. Legal requirements are met. The service will always support people to keep themselves and their belongings safe and secure. "	"The service has an inconsistent approach that sometimes puts people's safety, health or wellbeing at risk. There is an increased risk that people are harmed or there is limited assurance about safety. Regulations may or may not be met. "	"A service may have some areas of safe practice, but in general people are not safe. Normally some regulations are not met. "
**Effective**			
Outstanding	***Good***	***Requires improvement***	***Inadequate***
"Outcomes for people who use services are consistently better than expected when compared with other similar services. People's feedback about the effectiveness of the service describes it as exceptional and distinctive. "	"People's outcomes and feedback about the effectiveness of the service describes it as consistently good. "	"There is a lack of consistency in the effectiveness of the care and support that people receive. Regulations may or may not be met. "	"There are widespread and significant shortfalls in the care, support and outcomes that people experience. Normally some regulations are not met. "
**Caring**			
Outstanding	***Good***	***Requires improvement***	***Inadequate***
"People are truly respected and valued as individuals and are empowered as partners in their care by an exceptional and distinctive service. "	"People are supported and treated with dignity and respect, and are involved as partners in their care. "	"There are times when people do not feel well-supported or cared for, or their dignity is not maintained. The service is not always caring. Regulations may or may not be met. "	"People are not treated with compassion. There are breaches of dignity and significant shortfalls in the caring attitude of staff. Normally some regulations are not met. "
**Responsive**			
Outstanding	***Good***	***Requires improvement***	***Inadequate***
"Services are tailored to meet the needs of individual people and are delivered in a way to ensure flexibility, choice and continuity of care. "	"People's needs are met through the way services are organised and delivered. "	"Services do not always meet people's needs. Regulations may or may not be met. "	"Services are not planned or delivered in a way that meets people's needs. Normally some regulations are not met. "
**Well-led**			
Outstanding	***Good***	***Requires improvement***	***Inadequate***
"There are key characteristics that make leadership of the service exceptional and distinctive. The leadership, governance and culture are used to drive and improve high quality, person-centre care. "	"The service is consistently well-managed and led. The leadership, governance and culture promote the delivery of high-quality, person-centred care. "	"There is a lack of consistency in how well the service is managed and led. The leadership, governance and culture do not always support the delivery of high-quality, person-centred care. Regulations may or may not be met. "	"There are widespread and significant shortfalls in the way the service is led. Normally some regulations are not met. The delivery of high quality care is not assured by the leadership, governance or culture in place. "

Table A3: Type of inspections

Type of inspection	Scope	Trigger	Rating	Notice
**Comprehensive**	All five key questions	Scheduled or risk-based	Yes	Typically unannounced
**Focused**	Well-led and other relevant key questions related to information that triggered the inspection	Specific information or follow-up from previous inspection	Yes	Typically unannounced
**Targeted**	Will not involve a full domain, but will focus on the key lines of enquiry (1) within the relevant domain.	Particular risk or concern	No	Typically unannounced

More detail about the different inspection types can be found here (2)

Figure A1

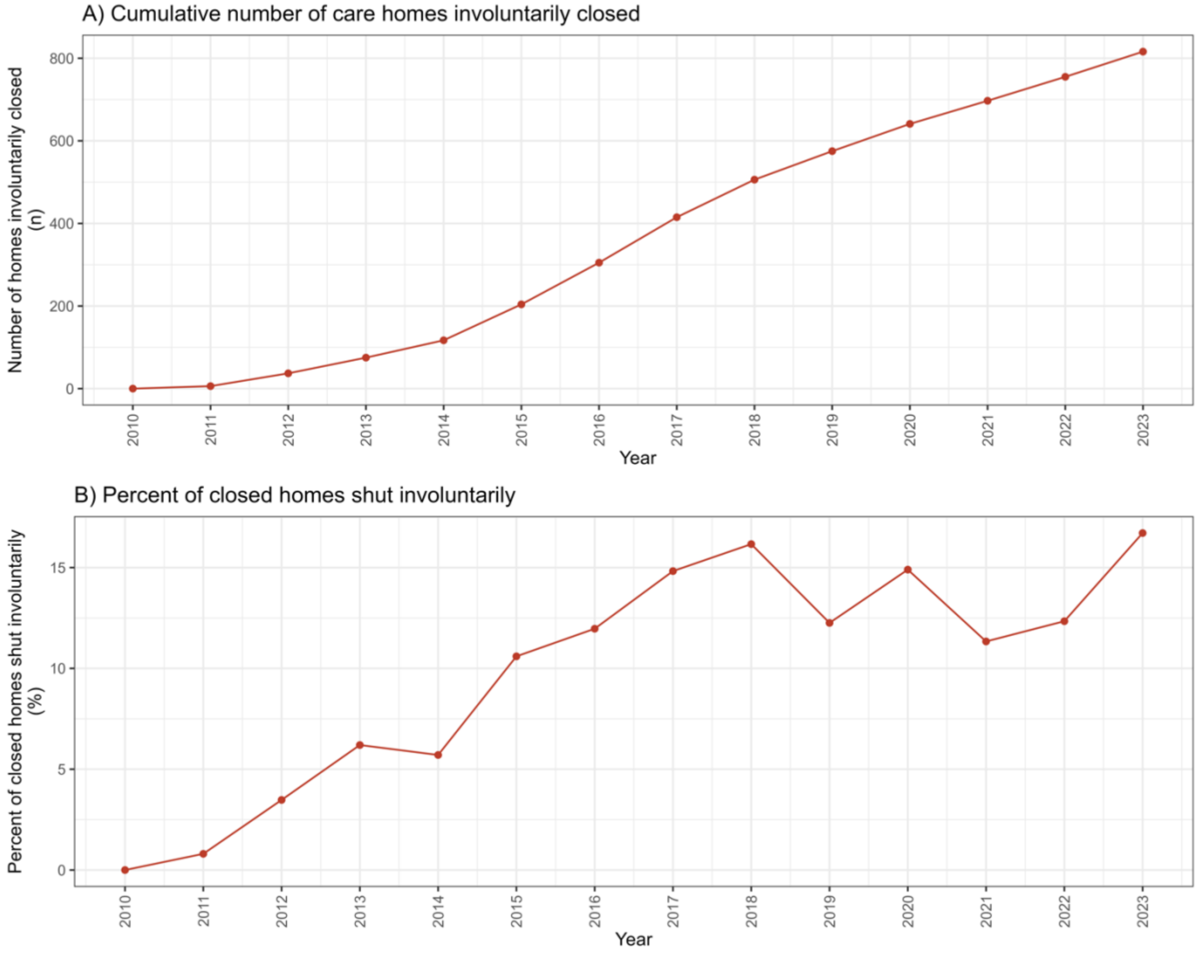

This figure visualises the yearly number of involuntary closures (panel A) and the proportion of closure types across ownership (panel B). Panel B reports the proportion of involuntary closures relative to voluntary closures that are not due to a change in provider ownership.

Figure A2: Homes that were involuntary closed without an inspection rating from the current inspection framework.

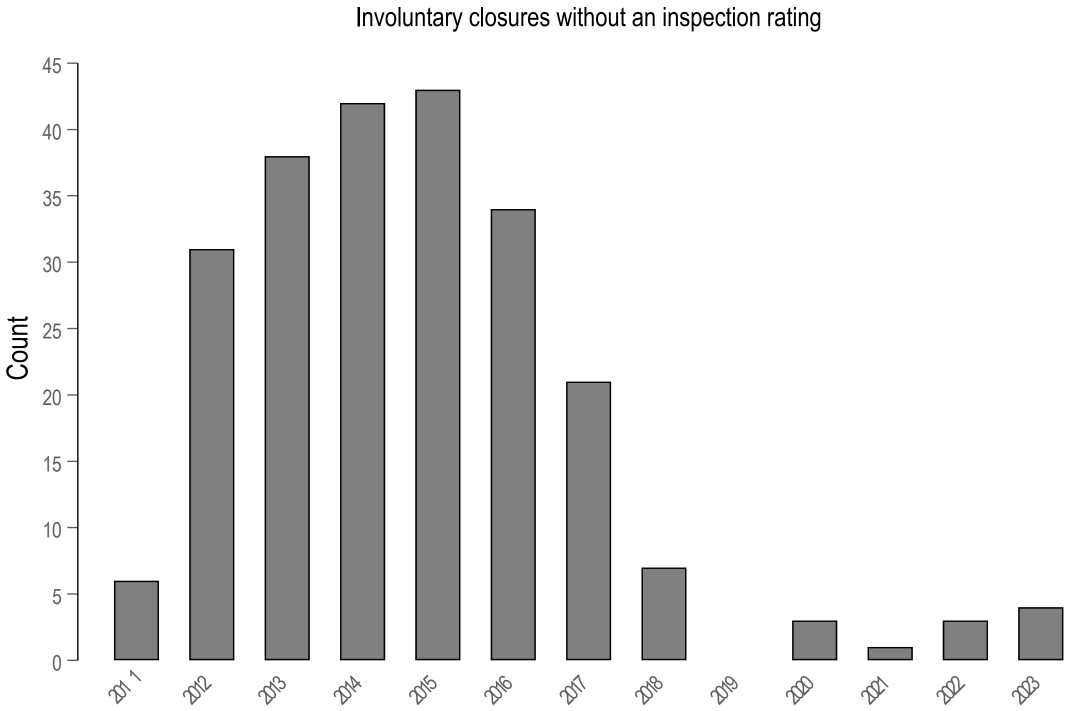

Figure A3: Development in the number and percentage of active for-profit, public, and third sector care homes and registered beds over time.

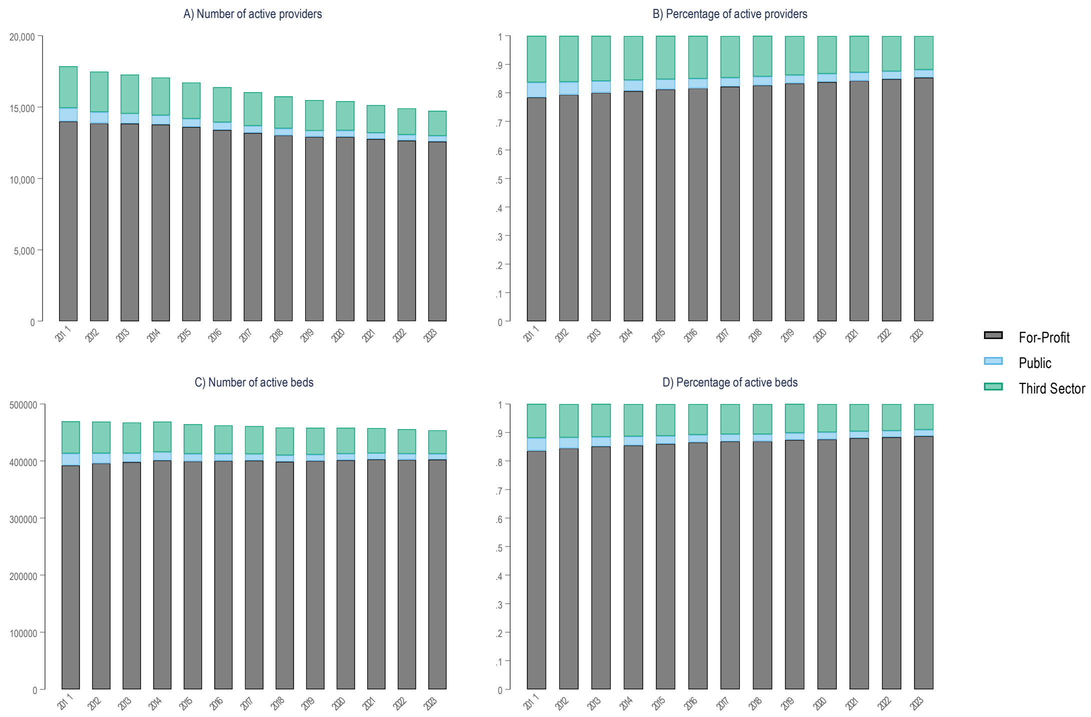

References

1. CQC. Key lines of enquiry for adult social care services - Care Quality Commission [Internet]. 2022 [cited 2024 Jan 4]. Available from: https://www.cqc.org.uk/guidance-providers/adult-social-care/key-lines-enquiry-adult-social-care-services

2. CQC. Types of inspection: adult social care services - Care Quality Commission [Internet]. 2022 [cited 2024 Jan 9]. Available from: https://www.cqc.org.uk/guidance-providers/adult-social-care/types-inspection-adult-social-care-services

3. CQC. How we monitor, inspect and regulate adult social care services - Care Quality Commission [Internet]. 2023 [cited 2024 Jan 4]. Available from: https://www.cqc.org.uk/guidance-providers/adult-social-care/how-we-monitor-inspect-regulate-adult-social-care-services

4. CQC. Key lines of enquiry, prompts and ratings characteristics for adult social care services [Internet]. 2017. Available from: www.cqc.org.uk/sites/default/files/20171020_adult_social_care_kloes_prompts_and_characteristics_final.pdf
